# Combined Addition of Microalgae and Probiotic Enhances Bacterial Community Network Stability, Water Quality, and Fish Growth in *Micropterus salmoides* Aquaculture

**DOI:** 10.3390/biology15070566

**Published:** 2026-04-01

**Authors:** Huimin Xu, Tianyu Zeng, Liping Qiu, Dandan Li, Longxiang Fang, Zhuping Liu, Yuhang Gao, Xi Chen, Limin Fan, Chao Song, Shunlong Meng

**Affiliations:** 1 Freshwater Fisheries Research Center, Chinese Academy of Fishery Sciences, Wuxi 214081, China; xuhuimin@ffrc.cn (H.X.);; 2 Laboratory of Quality & Safety Risk Assessment for Aquatic Products on Environmental Factors (Wuxi), Ministry of Agriculture and Rural Affairs, Wuxi 214081, China; 3 College of Fisheries and Life Sciences, Shanghai Ocean University, Shanghai 201306, China; 4 Wuxi Fisheries College, Nanjing Agricultural University, Wuxi 214081, China

**Keywords:** *Chlorella vulgaris*, *Bacillus subtilis*, free-living bacteria, particle-associated bacteria, co-occurrence network, functional prediction

## Abstract

Microalgae and probiotics are increasingly considered a promising approach for addressing environmental problems in freshwater aquaculture. However, how this approach affects the resident free-living (FL) and particle-associated (PA) bacterial communities that are essential in biogeochemical processes remains unclear. Here, we established a 60-day mesocosm aquaculture system for *Micropterus salmoides* to examine the effects of microalgal and probiotic addition on fish growth, water quality, and resident bacterial communities. Four treatments were included: *Chlorella vulgaris* addition (6.25 × 10^6^ cells/L), *Bacillus subtilis* addition (7.5 × 10^7^ CFU/L), their combined addition, and a control. Our results indicated that the combined addition significantly improved fish growth performance and reduced total and inorganic nitrogen concentrations than single additions. Moreover, the combined addition enhanced nitrogen- and carbon-related metabolic functions in FL fraction and increased host-associated functions in PA community. Taxa assigned with the classes Bacilli, Bacteroidia, Gammaproteobacteria, and Alphaproteobacteria showed predominant explanations for variations in nitrogen and phosphorus. Furthermore, the combined addition enhanced potential interactions among taxa across lifestyles and strengthened the bacterial community network complexity and stability. These findings provide new insights into the microbial mechanisms underlying microalgae–probiotic synergism and offer a scientific basis for sustainable microbial management in freshwater aquaculture.

## Simple Summary

Free-living and particle-associated bacteria jointly mediate matter and energy transformations in aquaculture systems, which are central for the functioning of microbial food web. Microalgae–probiotic additions are increasingly applied to mitigate nutrient accumulation in aquaculture and may exert important effects on both free-living and particle-associated bacterial groups; however, their ecological consequences for these resident bacterial communities remain poorly resolved. Here, we used controlled mesocosms stocked with largemouth bass to test single and combined additions of microalga (*Chlorella vulgaris*) and probiotic bacteria (*Bacillus subtilis*). The combined addition produced the strongest benefits, improving fish growth and reducing total nitrogen and dissolved inorganic nitrogen compared with single additions. It also influenced free-living and particle-associated bacterial communities in compositions and potential functions. Moreover, associations among bacterial groups became more interconnected under combined addition compared to single additions, indicating more resistance to disturbance. These findings clarify the microbial mechanisms underlying microalgae–probiotic synergism and support biologically based strategies to improve water quality and stability in freshwater aquaculture.

## 1. Introduction

Freshwater aquaculture has become an increasingly important component of global food production, providing affordable animal protein and supporting livelihoods worldwide. Aquaculture production has expanded rapidly in recent decades and is expected to continue increasing to meet growing food demand [1]. However, this rapid expansion is accompanied by environmental and ecological stresses, including water quality deterioration, nutrient accumulation, and disease outbreaks in cultured animals, which threaten both aquaculture productivity and sustainability [2]. In intensive aquaculture systems, large inputs of feed generate nutrient-rich environments that promote microbial growth. Microorganisms play crucial roles in aquaculture by mediating organic matter decomposition, nutrient transformation, and pathogen suppression, thereby maintaining water quality and ecosystem stability [3,4]. Within the water column, bacterial communities are generally divided into two ecological groups: free-living (FL) bacteria suspended in the water column, and particle-associated (PA) bacteria attached to organic particles [5,6]. FL bacteria can dominate nutrient transformations and dissolved organic matter utilization, whereas PA bacteria are primarily responsible for particulate organic matter decomposition and biofilm formation [7]. The typically high concentrations of organic particles in aquaculture environments create suitable microhabitats for FL and PA bacteria to colonize, thereby jointly shaping the functionality of the microbial food web. Despite the critical role of FL and PA bacteria, the diversity and compositions of FL and PA bacterial communities remain poorly understood in aquaculture environments.

To address the ecological challenges in aquaculture environments, various ecological strategies targeting the regulation of microbial communities have been employed, with the addition of microalgae and probiotic attracting particular attention [8,9]. Microalgae such as *Chlorella vulgaris* can assimilate dissolved nitrogen and phosphorus, produce oxygen and bioactive substances, and thereby improve water quality and promote the growth and health of cultured animals, which have been widely used in aquaculture as well as wastewater treatment [10,11]. Probiotics, particularly spore-forming *Bacillus* species, can facilitate organic matter degradation, suppress pathogens, and enhance host immunity, contributing to better water quality and animal performance [12,13,14]. More recently, microalgae–probiotic association has received increasing attention, as they exhibit synergetic relationships in carbon and nutrient cycling [15,16,17]. However, their combined application does not always result in consistent synergistic effects, as outcomes may vary with host species, culture conditions, dosage, and treatment duration. In closed, high-density aquaculture systems, the organic particles can be primarily derived from uneaten feed and animal excreta, together with plankton-derived materials [18,19]. Frequent addition of microalgae may shift the compositions of organic particles, thereby altering the compositions and metabolic activity of PA bacterial community [20,21]. Secondary metabolites produced by PA bacteria can, in turn, influence FL bacterial assemblages, with cascading effects on material fluxes and energy transfer within the microbial food web [22,23]. Moreover, the addition of microalgae can also affect bacterial communities through nutrient competition, carbon fixation, and the release of dissolved organic matter [15]. Similarly, the addition of probiotic can influence the resident FL and PA bacterial communities in aquaculture water by modulating organic matter degradation, altering nutrient fluxes, and releasing antimicrobial metabolites. However, most studies have only focused on the effects of microalgal or probiotic additions on water quality and animal growth, whereas their impacts on resident microbial communities remain poorly understood.

In aquaculture systems, bacterial taxa rarely act independently; instead, they are integrated into complex interaction networks [24]. These interactions, including mutualistic and competitive relationships among taxa with different lifestyles, are essential for biogeochemical processes [25]. Therefore, beyond compositional shifts, understanding microbial responses to the addition of microalgae and probiotic requires examining how microbial interactions are reorganized. Co-occurrence network analysis has been well established for describing potential interactions among microbial taxa [26,27]. Environments with greater substrate availability and niche heterogeneity generally harbor a more complex bacterial network, as diverse resources and microhabitats promote coexistence among taxa [28]. Microalgae exude diverse organic compounds, including polysaccharides, amino acids, and organic acids, that provide carbon and energy for heterotrophic bacteria [29,30]. Meanwhile, these organic compounds can be degraded by inoculated bacterial strains, forming spatially and chemically diverse microhabitats that enhance microbial niche differentiation [31]. Thus, it can be hypothesized that the combined addition of microalgae and probiotic would enhance substrate availability and microhabitat heterogeneity, thereby facilitating a more complex bacterial community network. Such a complex bacterial community network is likely to improve system resilience, nutrient turnover, and overall ecosystem stability. However, it remains unclear whether the bacterial community networks in aquaculture water shift toward higher complexity and stability following the combined addition of microalgae and probiotic.

Here, we conducted a mesocosm experiment to examine the responses of FL and PA bacterial communities to the addition of *Chlorella vulgaris* and *Bacillus subtilis*, individually and in combination, in largemouth bass (*Micropterus salmoides*) aquaculture systems. The largemouth bass possesses several advantages, including excellent flesh quality, the absence of intermuscular bones, rapid growth, a short culture cycle, strong adaptability, and high tolerance to handling. As a result, it has become one of the fastest-growing freshwater fish species in aquaculture production in China [32]. Specifically, we aimed to address the following questions: (1) Do the microalgal and bacterial additions improve aquaculture water quality and enhance the growth performance of cultured fish? (2) How do the compositions and potential functions of FL and PA bacterial communities respond to microalgal and bacterial additions? (3) How do microalgal and bacterial additions influence the co-occurrence network structure of bacterial communities in aquaculture water?

## 2. Materials and Methods

### 2.1. Experimental Design

The mesocosm experiment was carried out from July to September 2023. We used circular polyethylene tanks of 1000 L to mimic an aquaculture system. Each tank was filled with 800 L of tap water. The water was aerated with air pumps for 7 days before fish were stocked. Largemouth bass (*Micropterus salmoides*) were supplied by Jiangsu Zhongshui Dongze Agricultural Development Co., Ltd. (Wuxi, China). Only fish that were healthy, similar in size, active, and free of visible injuries were used.

The microalga used in this study was *Chlorella vulgaris*, obtained from the Freshwater Algae Culture Collection of the Chinese Academy of Sciences (FACH). The strain was grown in BG11 liquid medium in the laboratory to increase biomass. The final algal suspension had a cell density of 1 × 10^9^ cells/L. The selected probiotic was *Bacillus subtilis* from Shandong Zhonghaifa Biotechnology Co., Ltd. (Rizhao, China), with a viable count of 1 × 10^11^ CFU/g.

Four treatments were set up: addition of *C. vulgaris* (Alg_add), addition of *B. subtilis* (Bac_add), combined addition of both (Co_add), and a control without any addition (CK). In all treatments that received microalgae, *C. vulgaris* solution was added at 6.25 mL per liter of tank water. This gave an expected microalgal density of 6.25 × 10^6^ cells/L, which was based on our preliminary experiments. In all treatments that received the probiotic, *B. subtilis* powder was added at 0.75 mg per liter, giving an expected bacterial level of 7.5 × 10^7^ CFU/L. Microalgae and probiotics were added once every 10 days during the 60-day experiment, resulting in a total of six applications (days 0, 10, 20, 30, 40, and 50).

Each treatment had three independent tanks, and each tank contained 30 fish with an average body weight of 11.71 g. Fish were fed twice daily, at 09:00 and 17:00, with a commercial diet (Tongwei Co., Ltd., Wuxi, China; crude protein ≥ 46.0%, crude fiber ≤ 6.0%, crude fat ≥ 6.0%, crude ash ≤ 16.0%, total phosphorus ≥ 1.2%, lysine ≥ 2.3%, moisture ≤ 12.0%). The daily ration was about 3% of the total fish biomass in each tank, which was estimated from the weight of six fish randomly sampled from each group at each sampling time. Aeration was supplied continuously throughout the culture period.

To reduce disturbance to the microbial communities, no water exchange was carried out. After each sampling event, evaporative and sampling-related water losses were replenished to the original volume. The whole experiment lasted 60 days. The major operations and their temporal sequence on days 0, 10, 20, 30, 40, 50, and 60 are summarized in Figure S1.

During the experiment, dissolved oxygen, pH, and water temperature were monitored as background environmental variables. Dissolved oxygen remained within 7.12~7.74 mg/L, pH ranged from 7.5 to 8.2, and water temperature ranged from 25.3 to 32.1 °C.

All experimental protocols were approved by the Ethics Committee of the Freshwater Fisheries Research Centre of the Chinese Academy of Fishery Sciences (FFRC, Wuxi, China) (Approval Number: LAECFFRC-2023-05-18).

### 2.2. Sample Collection

Water samples were collected on days 20 and 60. Sampling on day 20 was performed immediately before the scheduled addition on that day, whereas day 60 sampling was conducted at the end of the experiment before termination of the culture trial. Samples were placed in 1 L polyethylene bottles and stored at 4 °C for further water quality measurements. The 1 L water samples were also collected for characterizations of free-living (FL) and particle-associated (PA) bacterial community compositions. Biomass of FL and PA bacterial communities were collected according to the protocol of Zhao et al. (2017) [33]. Both FL and PA filters were preserved at −80 °C until DNA extraction.

Throughout the experiment, the survival rate of largemouth bass in all aquaculture systems remained at 100%. Body weights of largemouth bass in the 12 aquaculture systems across the four treatments were measured both before and after the experiment. Weight gain rates and specific growth rates were calculated to evaluate the growth performance of the cultured organisms.

### 2.3. Measurements of Water Quality

Unfiltered water samples were used to measure the total nitrogen (TN) and total phosphorus (TP). Furthermore, water samples were filtered through 0.45 μm pore size polycarbonate membrane filter (47 mm diameter; Millipore, Billerica, MA, USA). Ammonium nitrogen (NH_4_^+^-N), nitrate nitrogen (NO_3_^−^-N), and nitrite nitrogen (NO_2_^−^-N) were determined in the filtrate according to national standard methods.

### 2.4. DNA Extraction, PCR, and High-Throughput Sequencing

The DNA was extracted from a total of 48 samples using the E.Z.N.A. D5525-01 Water DNA Kit (Omega Bio-tek, Norcross, GA, USA). The V3–V4 region of the 16S ribosomal RNA gene of the bacteria was amplified using primers 338F (5′-ACTCCTACGGGAGGCAGCAG-3′) and 806R (5′-GGACTACHVGGGTWTCTAAT-3′) in a polymerase chain reaction (PCR) [34]. The PCR mixture and thermal cycling conditions were consistent with the existing procedures reported in our previous study [35]. For each sample, PCR was performed three times. The Illumina paired-end library was constructed, and the amplicons were then sequenced using the Illumina Miseq PE 300 platform (San Diego, CA, USA) at Majorbio Bio-Pharm Technology Co. Ltd. (Shanghai, China). The raw data are accessible in NODE (https://www.biosino.org/node) through the URL: https://www.biosino.org/node/experiment/detail/OEX00032700 (accessed on 3 November 2025).

### 2.5. Sequence Processing

Paired-end reads for each sample were processed using QIIME 2 (v2021.8) [36]. Sequence reads were quality-filtered and denoised using the q2-dada2 plugin, which yielded ASV-level feature tables (read counts) [37]. Taxonomic information of ASVs was obtained according to the SILVA SSU 138.2 database [38]. ASV feature tables were filtered to remove unclassified taxa, mitochondrial and chloroplast ASVs, and low-abundance ASVs (<0.0005%). Then, ASVs were aligned and a phylogenetic tree was constructed. The number of observed ASVs and Pielou’s evenness were calculated as alpha diversity. Bray–Curtis distance matrix was also generated. Sequences were finally rarefied to 37,392 reads per sample, yielding 3246 bacterial ASVs for downstream analyses.

### 2.6. Statistical Analyses

Differences in growth performance indices among treatments were tested using one-way ANOVA followed by Duncan’s post hoc test. A *t*-test was applied to assess differences in environmental parameters between day 20 and day 60. For each sampling time point (day 20 and day 60), one-way ANOVA combined with Duncan’s post hoc test was further used to examine treatment effects on environmental variables. A three-way ANOVA was performed to evaluate the effects of lifestyle, treatment, and samplingtime pointt on alpha diversity. Differences in bacterial community composition across lifestyle, treatment, and samplintime pointnt were analyzed using PERMANOVA. Principal coordinate analysis (PCoA) was conducted to visualize the compositional differences among samples in FL and PA bacterial communities. We used the ‘DESeq2’ package in R to identify significantly enriched or depleted ASVs in the Alg_add, Bac_add, and Co_add treatments relative to the control group. DESeq2 applies a model based on the negative binomial distribution to estimate variance-mean dependence and test for differential abundance of microbial taxa between groups [39]. ASVs with an adjusted *p*-value (false discovery rate, FDR) below 0.05 and an absolute log2 fold change greater than 1 was detected as significantly enriched or depleted in the Alg_add, Bac_add, and Co_add treatments compared to CK treatment. The potential functions of the enriched and depleted ASVs were predicted according to the Functional Annotation of Prokaryotic Taxa (FAPROTAX) database [40]. Random forest analyses were conducted to evaluate the individual contributions and significance of ASVs that were significantly enriched relative to the CK treatment in explaining changes in TN, TP, NH_4_^+^-N, NO_3_^−^-N, and NO_2_^−^-N.

Co-occurrence network analyses were conducted for the bacterial communities of the four treatments using community data derived from both lifestyles (FL and PA) and sampling time points (days 20 and 60). ASVs with a relative abundance threshold of ≥0.01% and an occurrence frequency of ≥30% were involved in the construction of networks. The SparCC method was used to infer correlations among the filtered ASVs, retaining associations with sparcc.R ≥ 0.6 and sparcc.*p* < 0.05 [41]. Correlations between environmental variables and ASVs were determined using Spearman’s rank correlation analysis, with only strong and significant correlations (r ≥ 0.85, *p* < 0.05) retained for network construction. In each network, the package ‘DESeq2’ was used to identify differences in relative abundance of each ASV between the FL and PA bacterial communities in R (version 4.5.1), thereby defining the lifestyle preference of each ASV. ASVs that were significantly enriched in PA relative to FL were classified as having a PA lifestyle preference (i.e., belonging to the ‘PAPL’ cluster), whereas ASVs that were significantly depleted in PA relative to FL were classified as having an FL lifestyle preference (i.e., belonging to the ‘FLPL’ cluster). ASVs showing no significant differences between FL and PA were considered to have no specific lifestyle preference (i.e., belonging to the ‘others’ cluster). Network visualizations were generated using Gephi (v0.9.2). Topological properties including modularity, clustering coefficient, average path length, network diameter, and average degree were calculated using the package ‘igraph’ in R (version 4.5.1) [42]. For each empirical network, 1000 size-matched random networks were generated using the R package ‘igraph’, and network indices were computed for each random network individually. Z-tests were then used to assess whether network indices differed significantly between empirical and random networks. Network robustness and vulnerability were quantified for all empirical networks following Yuan et al. (2021) [43].

## 3. Results

### 3.1. Growth Performance of the Cultured Fish

We analyzed the growth performance of largemouth bass across different treatments (Table 1). Compared with the CK treatment, the Alg_add, Bac_add, and Co_add treatments showed a general trend of improved weight gain rate and specific growth rate. Compared with the CK treatment, the Alg_add, Bac_add, and Co_add treatments increased the weight gain rate by 15.5%, 21.2%, and 32.3%, respectively. Similarly, the specific growth rate increased by 7.6%, 8.6%, and 12.6%, respectively. Furthermore, the Co_add treatment exhibited significantly higher final body weight, weight gain rate, and specific growth rate than the CK (*p* < 0.05). In contrast, the Alg_add and Bac_add treatments did not differ significantly from the CK (*p* > 0.05).

### 3.2. Water Quality Parameters

**Figure 1:**
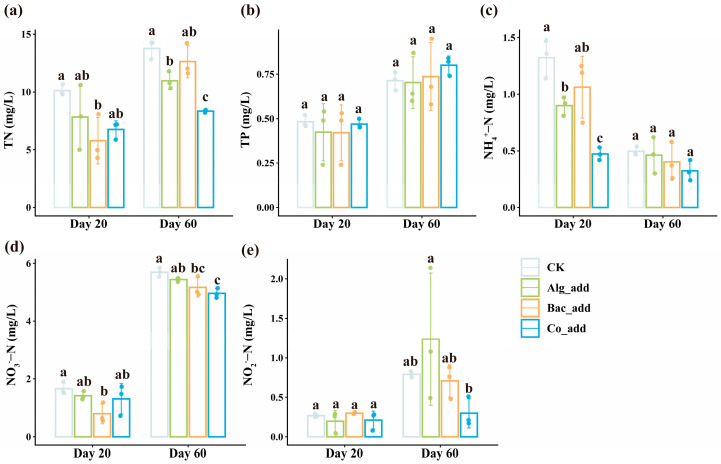
Differences in water quality parameters of aquaculture systems across treatments using ANOVA tests with Duncan’s post hoc tests. (**a**) TN, total nitrogen; (**b**) TP, total phosphorus; (**c**) NH_4_^+^-N, ammonia nitrogen; (**d**) NO_3_^−^-N, nitrate nitrogen; (**e**) NO_2_^−^-N, nitrite nitrogen; CK, control treatment; Alg_add, treatment with single addition of microalgae; Bac_add, treatment with single addition of probiotic; Co_add, treatment with combined addition of microalgae and probiotic. Different letters indicate significant differences across treatments on day 20 or day 60 (*p* < 0.05). Bars represent mean ± SD, and raw data points are shown (n = 3 per treatment at each sampling time point).

Water quality parameters of the aquaculture systems showed differences across treatments at the two sampling time points (Figure 1). Overall, the concentrations of TN, TP, NO_3_^−^-N, and NO_2_^−^-N of all aquaculture systems were higher on day 60 than those on day 20, whereas the concentration of NH_4_^+^-N showed the contrast pattern (*t*-test, *p* < 0.001 for all tests). TN concentrations remained consistently highest in the CK treatment throughout the experiment (Figure 1a). On day 60, the TN concentration of Co_add treatment was significantly lower than the other three treatments. Furthermore, TN concentration of Alg_add treatment was significantly lower than CK and Bac_add treatments, whereas CK and Bac_add treatments exhibited similar TN levels. However, TP concentrations were comparable across all treatments at both time points (Figure 1b). On both days 20 and 60, NH_4_^+^-N concentrations consistently remained the highest in the CK and the lowest in the Co_add (Figure 1c). However, significant differences across the treatments were observed only on day 20, while no significant differences in NH_4_^+^-N concentrations were detected among the four treatment groups on day 60. Similarly, at both sampling time points, NO_3_^−^-N concentrations remained consistently highest in the CK treatment (Figure 1d). On day 20, the NO_3_^−^-N concentration in the Bac_add treatment was significantly lower than in the other treatments. By day 60, NO_3_^−^-N concentrations in both the Bac_add and Co_add treatments were significantly lower than in the CK treatment. As for NO_2_^−^-N, no significant differences were observed across the treatments on day 20 (Figure 1e). However, on day 60, NO_2_^−^-N concentration in the Co_add treatment was significantly lower than in the other treatments.

### 3.3. Diversity and Structure of FL and PA Bacterial Communities

The number of observed ASVs was used to represent bacterial community richness, while Pielou’s evenness was used to indicate community evenness. Three-way ANOVA tests revealed that both richness and evenness differed significantly between the two lifestyles, but not among the different treatments or time points (Table S1). Both the richness (454 ± 56 for PA and 282 ± 109 for FL) and evenness (0.74 ± 0.04 for PA and 0.52 ± 0.14 for FL) of PA bacterial communities were significantly higher than those of FL bacterial communities (*t*-test, *p* < 0.001). Furthermore, greater variability in richness and evenness across different treatments and time points was observed in the FL communities, as indicated by their higher standard deviation values.

**Figure 2:**
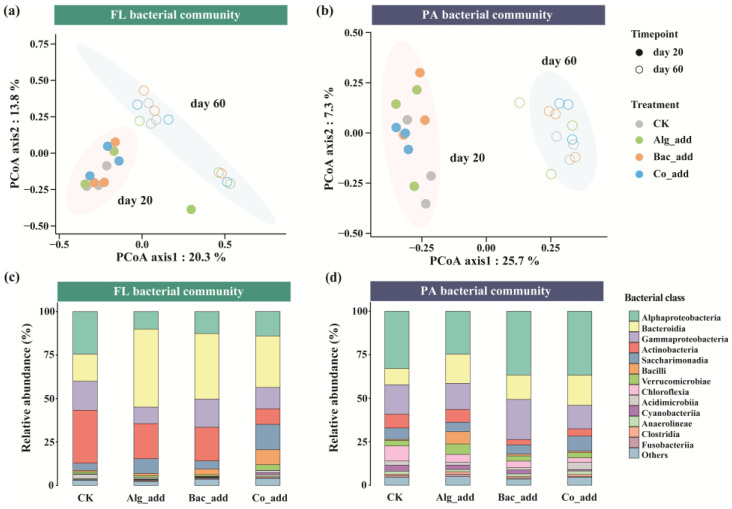
Structure and compositions of free-living (FL) and particle-associated (PA) bacterial communities in the aquaculture systems with different treatments. (**a**,**b**) Principal coordinate analysis (PCoA) of the bacterial communities based on Bray–Curtis distance. (**c**,**d**) Taxonomic compositions of the FL and PA bacterial communities of the aquaculture systems with different treatments at the class level. Only the dominant classes (relative abundance >0.5%) are represented. CK, control treatment; Alg_add, treatment with single addition of microalgae; Bac_add, treatment with single addition of probiotic; Co_add, treatment with combined addition of microalgae and probiotic.

PERMANOVA based on Bray–Curtis distances revealed that lifestyle and time point were the primary factors influencing bacterial community dissimilarity, explaining a total of 23% of the community dissimilarity (Table 2). In addition, the treatment factor showed a significant but weaker influence (R^2^ = 0.07, F = 1.4, *p* < 0.05). Notably, the interaction between lifestyle and sampling time was also significant (R^2^ = 0.06, F = 3.5, *p* < 0.001), whereas the interactions among lifestyle and treatment, treatment and sampling time, and the three factors together were not statistically significant. The PCoA plots showed that the first two components (PCoA1 and PCoA2) explained a total of 34.1% and 33.0% of the variance in the FL and PA bacterial community compositions across different treatments and time points, respectively (Figure 2a,b). Furthermore, FL and PA bacterial community compositions were more influenced by sampling time rather than treatments.

The taxonomic compositions of FL and PA bacterial communities varied across lifestyles and treatments (Figure 2c,d). FL and PA bacterial communities were both dominated by the classes Alphaproteobacteria, Bacteroidia, Gammaproteobacteria, Actinobacteria, and Sacharimonadia, each exhibiting an average relative abundance greater than 5%. However, the relative abundances of these classes differed between the FL and PA communities. In the FL bacterial community, Bacteroidia accounted for more than 30% on average, followed by Actinobacteria (19.7%) and Alphaproteobacteria (15.3%) (Figure 2c). In the PA bacterial community, Alphaproteobacteria showed the highest average relative abundance (32.7%), followed by Gammaproteobacteria (17.1%) and Bacteroidia (14.4%). In addition, the relative abundances of Bacteroidia in both FL and PA communities were higher in the Alg_add, Bac_add, and Co_add treatments than in the CK, with a greater increase observed in the FL bacterial community than in the PA community. The classes Saccharimonadia and Bacilli showed higher relative abundances in the Co_add treatment than in the other three treatments. In the PA bacterial community, Bacilli and Verrucomicrobiae exhibited higher average relative abundances in the Alg_add treatment than in the other treatments. The classes Saccharimonadia and Acidimicrobiia had the highest average relative abundances in the Co_add treatment, while Cyanobacteriia showed the lowest abundance overall (Figure 2d). Additional taxonomic composition data for the FL and PA bacterial communities at the phylum and genus levels are provided in Figures S2 and S3 of the Supplementary Materials.

### 3.4. Identification of Differential Taxa of Different Treatments and Their Ecological Characteristics in FL and PA Bacterial Communities

**Figure 3:**
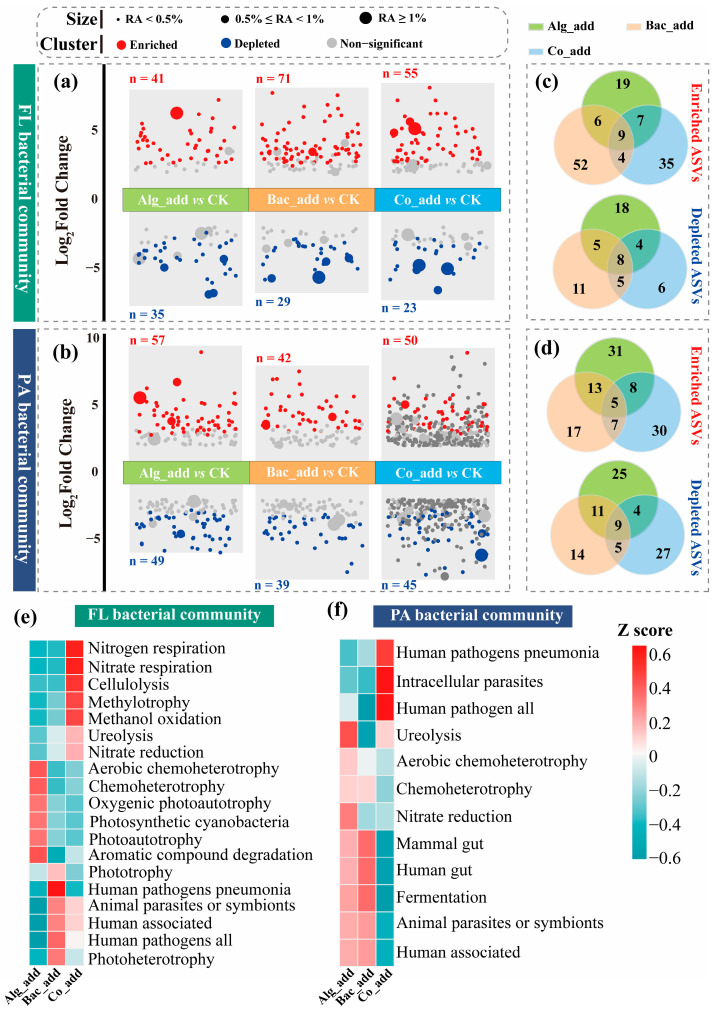
Differential changes in bacterial community composition under different microbial treatments and potential functions of enriched taxa. (**a**,**b**) Volcano plots showing significant changes in amplicon sequence variants (ASVs) in the free-living (FL, panel (**a**)) and particle-associated (PA, panel (**b**)) bacterial communities across the Alg_add, Bac_add, and Co_add treatments compared to the CK, with enriched ASVs in red and depleted ASVs in blue. (**c**,**d**) Venn diagrams illustrating the number of shared and unique differentially abundant ASVs for FL (panel (**c**)) and PA (panel (**d**)) bacterial communities across treatments. (**e**,**f**) Heatmaps showing the relative abundance (standardized by Z-score) of predicted functional profiles of enriched ASVs in FL (**e**) and PA (**f**) bacterial communities under different treatments based on Functional Annotation of Prokaryotic Taxa (FAPROTAX) database. CK, control treatment; Alg_add, treatment with single addition of microalgae; Bac_add, treatment with single addition of probiotic; Co_add, treatment with combined addition of microalgae and probiotic. TN, total nitrogen; TP, total phosphorus; NH_4_^+^-N, ammonia nitrogen; NO_3_^−^-N, nitrate nitrogen; NO_2_^−^-N, nitrite nitrogen. Non-significant results are not displayed in (**e**).

The addition of microalgae and probiotic significantly altered the composition of FL and PA bacterial communities in aquaculture water. ASVs that were significantly enriched or depleted relative to the CK treatment were identified in the Alg_add, Bac_add, and Co_add treatments (Figure 3a,b). In the Alg_add treatment, both the number of enriched and depleted ASVs were higher in the PA bacterial community than FL bacterial community. In contrast, in the Bac_add and Co_add treatments, the number of enriched ASVs was markedly lower in the PA fraction than in the FL fraction, whereas more ASVs were significantly depleted in the PA bacterial community than in the FL bacterial community.

We further analyzed the overlap of enriched and depleted ASVs among treatments (Figure 3c,d). In the FL bacterial community, 73.2% and 63.6% of the enriched ASVs were unique to the Bac_add and Co_add treatments, while only 46.3% were unique to the Alg_add treatment. For depleted ASVs in the FL community, the trend was opposite. The Alg_add treatment had the highest proportion of unique depleted ASVs (51.4%), whereas the Bac_add and Co_add treatments had lower proportions (37.9% and 26.1%). In the PA bacterial community, 54.4% and 60% of the enriched ASVs were unique to the Alg_add and Co_add treatments, both higher than the 40.5% observed in the Bac_add treatment. Similarly, the proportions of unique depleted ASVs in the Alg_add and Co_add treatments were 51.1% and 60%, which also exceeded that in the Bac_add treatment (35.9%).

The predicted functions of these enriched ASVs differed notably between the FL and PA bacterial communities (Figure 3e,f). The enriched ASVs of the FL bacterial community were characterized by a higher relative abundance of nitrogen- and carbon-related metabolic functions, while those of the PA bacterial community contained more host-associated and heterotrophic functions. In the FL bacterial community, functions related to nitrogen metabolism, including nitrogen respiration and nitrate respiration, and nitrate reduction, were more abundant in the Co_add treatment compared to the Alg_add and Bac_add treatments (Figure 3e). Functions associated with carbon transformation, such as cellulolysis, methylotrophy, and methanol oxidation, also showed higher relative abundance in the Co_add treatment. Photoautotrophic and phototrophic functions, including oxygenic photoautotrophy and photosynthetic cyanobacteria, were mainly observed in the Alg_add treatment. In addition, heterotrophic functions, such as chemoheterotrophy, aerobic chemoheterotrophy, and aromatic compound degradation, were also detected with higher relative abundance in the Alg_add treatment. Functions associated with human or animal hosts, such as human pathogens pneumonia and animal parasites or symbionts, were detected with a higher relative abundance in the Bac_add treatment compared to the other two treatments, although the relative abundances of these functions were at relatively lower levels than in the PA bacterial community. In the PA bacterial community, the predicted functions were dominated by functions related to host association, including human pathogens pneumonia, intracellular parasites, and mammal or human gut, which showed higher relative abundance in the Co_add than the other two treatments (Figure 3f). Furthermore, ureolysis, nitrate reduction, chemoheterotrophy, and aerobic chemoheterotrophy were more abundant in the Alg_add treatment than the other two treatments.

**Figure 4:**
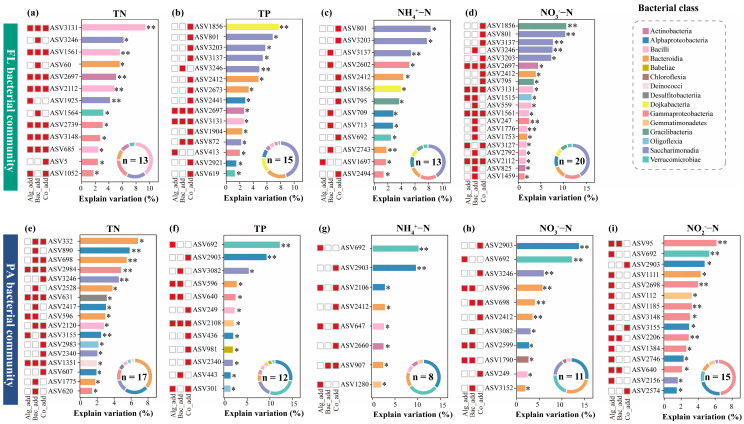
Contributions of bacterial ASVs significantly enriched in free-living (FL) and particle-associated (PA) bacterial communities under Alg_add, Bac_add, and Co_add treatments to explaining variation in environmental factors of aquaculture water based on random forest models (n = 6 for each treatment). The length of the bars represents the explained variation, and the color of each bar indicates the bacterial class of the ASV. Red squares on the left show whether the ASV was significantly enriched in the respective treatment group. Circular charts summarize the relative contributions of different bacterial classes to explaining variations in each environmental factor, with ‘n’ denoting the total number of ASVs included. Panels (**a**–**d**) correspond to environmental factors TN (**a**), TP (**b**), NH_4_^+^-N (**c**), and NO_3_^−^-N (**d**) for the FL bacterial community, while panels (**e**–**i**) show the same factors for the PA bacterial community: (**e**) TN, (**f**) TP, (**g**) NH_4_^+^-N, (**h**) NO_3_^−^-N, (**i**) NO_2_^−^-N. CK, control treatment; Alg_add, treatment with single addition of microalgae; Bac_add, treatment with single addition of probiotic; Co_add, treatment with combined addition of microalgae and probiotic. TN, total nitrogen; TP, total phosphorus; NH_4_^+^-N, ammonia nitrogen; NO_3_^−^-N, nitrate nitrogen; NO_2_^−^-N, nitrite nitrogen. Non-significant results are not displayed. * *p* < 0.05; ** *p* < 0.01.

We further used random forest models to evaluate the contribution of individual enriched ASVs to explaining environmental variations in aquaculture water (Figure 4). Based on the explanatory contributions of enriched ASVs and their taxonomic affiliations, we summarized the relative importance of different bacterial classes in the models of various environmental factors (Figure 4). In the FL bacterial community, ASVs belonging to the classes Bacilli, Saccharimonadia, Bacteroidia, and Gammaproteobacteria were significantly enriched across all three treatment groups and played important roles in influencing TN, TP, NH_4_^+^-N, and NO_3_^−^-N (Figure 4a–d). In the PA bacterial community, enriched ASVs belonging to the classes Alphaproteobacteria, Verrucomicrobiae, Bacteroidia, and Gammaproteobacteria played important roles in influencing TN, TP, NH_4_^+^-N, NO_3_^−^-N, and NO_2_^−^-N (Figure 4e–i). Furthermore, in FL bacterial community, ASVs with significant effects on total TN were relatively evenly distributed among the Alg_add, Bac_add, and Co_add treatments (Figure 4a). ASVs influencing TP and NH_4_^+^-N were mainly enriched under the Co_add treatment, whereas those associated with NO_3_^−^-N were predominantly found in both the Bac_add and Co_add treatments (Figure 4b–d). In the PA bacterial community, ASVs contributing to TN and NO_3_^−^-N variations were primarily enriched under the Bac_add and Co_add treatments (Figure 4e,h). ASVs related to TP and NH_4_^+^-N occurred mainly in the Co_add treatment, while those associated with NO_2_^−^-N were enriched in both the Alg_add and Co_add treatments (Figure 4f,g,i).

### 3.5. Co-Occurrence Patterns of Bacterial Communities of Different Treatments

**Figure 5:**
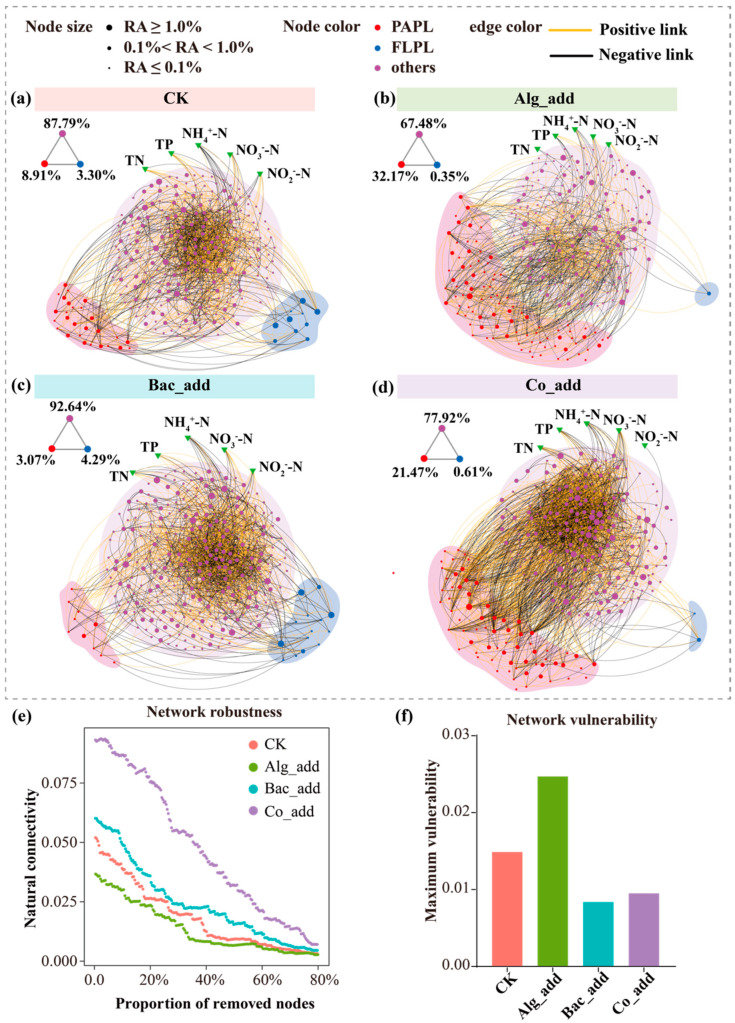
Co-occurrence network analyses. (**a**–**d**) Co-occurrence patterns of bacterial ASVs and environmental variables. Circular nodes represent bacterial ASVs, and triangular nodes represent environmental variables. For circular nodes, different colors indicate the lifestyle preferences of ASVs, and node size is proportional to their relative abundance. Numbers adjacent to the symbols denote the proportion of ASVs with different lifestyle preferences. Yellow and black lines represent positive and negative correlations, respectively. (**e**,**f**) Network stability is reflected in network robustness and vulnerability. CK, control treatment; Alg_add, treatment with single addition of microalgae; Bac_add, treatment with single addition of probiotic; Co_add, treatment with combined addition of microalgae and probiotic.

Four treatment-specific co-occurrence networks were built, each including FL and PA bacterial communities (Figure 5a–d). The topological characteristics of these networks are summarized in Table 3. Modularity, clustering coefficient, network diameter, and average path length differed significantly between the four empirical bacterial community networks and their corresponding random networks, indicating non-random network structure (Table 3). Furthermore, comparison of the topological parameters revealed that the Alg_add treatment had the lowest average degree, followed by the CK and Bac_add treatments, whereas the Co_add treatment exhibited the highest average degree, far exceeding that of the other networks. In addition, the modularity of all four networks was below 0.4. The Co_add treatment also showed a lower network diameter and shorter average path length than the other treatments, but a higher clustering coefficient.

We also found that the addition of microalgae influenced the lifestyle preferences of bacterial ASVs in the co-occurrence networks (Figure 5a–c). ASVs with no clear lifestyle preference predominated across all four treatments, ranging from 67.48% to 92.64%. In the CK network, ASVs belonging to ‘PAPL’ (particle-associated lifestyle preference) and ‘FLPL’ (free-living lifestyle preference) clusters accounted for 8.91% and 3.30% of the nodes, respectively (Figure 5a). In contrast, 32.17% of nodes in the Alg_add network and 21.47% in the Co_add network belonged to the ‘PAPL’ cluster (Figure 5a,d), higher than in the CK treatment, whereas the proportion of nodes assigned to the ‘FLPL’ cluster was less than 1%. In the Bac_add network, ASVs assigned to the ‘PAPL’ cluster accounted for only 3.07% (Figure 5c), which was lower than in the CK treatment, while ‘FLPL’ ASVs reached 4.29%, the highest among all treatments. Furthermore, significant associations with environmental factors were detected only for ASVs belonging to ‘others’ (i.e., ASVs with no clear lifestyle preference) cluster (Figure 5a–c).

Furthermore, in the CK network, most links occurred among ‘others’ nodes, with relatively few links connecting ‘others’ to ‘PAPL’ or ‘FLPL’ nodes (Figure 5a). In the Alg_add network, the number of links between the nodes affiliated with ‘others’ and ‘PAPL’ clusters increased, and denser intra-group connections were observed among ‘PAPL’ nodes compared with the CK network (Figure 5a,b). The Bac_add network showed fewer interconnections of ASVs across lifestyles, with most links concentrated within the ‘others’ cluster and limited connections between ‘others’ and the other two clusters (Figure 5c). In contrast, the Co_add network exhibited the highest degree of interconnection, characterized by numerous links between the nodes of ‘others’ and ‘PAPL’ clusters, along with dense intra-group connections within the ‘others’ cluster (Figure 5d).

Network stability was further assessed by robustness and vulnerability (Figure 5e,f). The Alg_add network exhibited the lowest robustness, followed by the CK and Bac_add networks, whereas the Co_add network was substantially more robust than the other three. In contrast, vulnerability was highest in the Alg_add network, followed by CK, Bac_add, and Co_add.

## 4. Discussion

### 4.1. The Responses of Bacterial Communities to the Addition of Microalgae and Probiotic Differed Between Free-Living and Particle-Associated Lifestyles

In complex aquatic ecosystems, the PA taxa colonize particle surfaces or aggregates characterized by heterogeneous and resource-rich microhabitats, whereas FL taxa inhabit the surrounding water column where nutrients are predominantly dissolved [44]. FL and PA bacteria are two vital components of bacterial communities in aquaculture water environments, yet they have been largely overlooked in previous studies. In the present study, we demonstrated variations in the diversity, compositions, and potential functions of FL and PA bacterial communities within aquaculture water columns across different treatments with single or combined additions of microalgae and probiotic. The PA bacterial communities exhibited higher richness and evenness than the FL fraction across all treatments and time points, which were consistent with results from previous research on FL and PA bacterial communities in lakeshore ecosystems [45]. In our experimental systems, continuous inputs of feed and microalgae increased the organic matter content, thereby providing abundant resources for the PA bacterial community and supporting its higher diversity [5].

Although the addition of microalgae and probiotic had limited overall effects on the FL and PA bacterial communities, distinct bacterial taxa were significantly enriched in the treatments with single and combined additions of microalgae and probiotic. In the FL bacterial community, the majority of the enriched ASVs were unique to the treatments involved with the addition of probiotic. However, in the PA bacterial community, the majority of the enriched ASVs were unique to the treatments involved with the addition of microalgae. In the FL bacterial community, the enrichment of specific taxa under probiotic addition treatments (Bac_add and Co_add) may be due to *Bacillus subtilis*. They can release low-molecular-weight organic carbon (LMW-DOC) and fluorescent compounds during metabolism, which could further influence the FL bacterial community dependent on dissolved organic matter [46,47,48]. On the other hand, in the PA bacterial community, microalgal additions were more influential in enriching bacterial taxa than the single addition of probiotic. This can be explained by the fact that PA communities are typically associated with particles, which provide a stable and nutrient-rich environment for bacteria. Microalgae can release organic compounds through exudation that can be utilized by bacteria attached to particles, facilitating their growth [21]. Moreover, the microalgae themselves may serve as substrates for PA bacteria, fostering the growth of taxa that specialize in the degradation of algal-derived organic matter [6].

Functional prediction showed that the enriched taxa differed in their potential functions between the FL and PA communities after microalgal and probiotic addition. In the FL bacterial community, the combined addition markedly enhanced metabolic functions related to carbon and nitrogen compounds with low molecular weight, including nitrate reduction, nitrogen respiration, cellulolysis, and methylotrophy. Wang et al. (2022) also found that the abundance of denitrification genes in the recirculating aquaculture system with the combined addition of algae and bacteria was higher than in the control group, which is associated with nitrate reduction and nitrogen respiration [17]. Our findings further highlighted that the bacterial taxa enriched following the combined addition of microalgae and probiotic, primarily related to the metabolism of carbon and nitrogen compounds with low molecular weight, predominantly exhibit the FL lifestyle. The enrichment of chemoheterotrophic functions under the Alg_add treatment was detected in both FL and PA bacterial communities, which were more enriched under the single addition of microalgae (Alg_add) than under the combined addition (Co_add). This pattern likely reflects the strong stimulation of heterotrophic metabolism by algal exudates that provide labile organic carbon for bacterial respiration [49]. In the Co_add treatment, however, the introduction of additional bacterial strains may have altered substrate composition and metabolic partitioning, promoting more diverse processes such as nitrogen transformations and methylotrophy. As a result, the relative contribution of chemoheterotrophic functions was diluted, even though overall metabolic activity was likely enhanced. However, host-associated functions were enriched in the PA community after both single and combined additions of microalgae and probiotic. This pattern suggests closer associations between microalgae and surface-attached bacteria. The surfaces of microalgal cells and microalgal-derived particles represent microhabitats that are rich in organic matter, signaling molecules, and adhesive matrices such as extracellular polymeric substances [50]. These features create microenvironments that promote bacterial attachment, colonization, and metabolic interactions, which are similar to host surfaces [51]. However, it should be noted that host-associated functional categories predicted by FAPROTAX are inferred based on gene similarity to known reference genomes [40]. Therefore, their enrichment could refer to shifts in functional capacity or lifestyle toward more surface-associated niches rather than an actual increase in pathogenic bacteria.

Random forest results showed that different enriched bacterial taxa explained environmental variations between the FL and PA communities. In the FL fraction, the classes Bacilli, Saccharimonadia, Bacteroidia, and Gammaproteobacteria showed the significant contributions to environmental variations. In contrast, the PA fraction was dominated by enriched Alphaproteobacteria, Verrucomicrobiae, Bacteroidia, and Gammaproteobacteria as top contributors to environmental variations. Bacilli and Saccharimonadia mainly drive the rapid turnover of labile organic matter in the dissolved phase [52,53], while Alphaproteobacteria and Verrucomicrobiae specialize in particle-associated degradation of complex polysaccharides and promote coupled carbon–nitrogen cycling through biofilm-based interactions [54,55]. These results suggest that the distinct responses of the FL and PA communities played complementary roles in organic matter decomposition and nutrient turnover under microalgal and bacterial addition.

### 4.2. The Combined Addition of Microalgae and Probiotic Enhanced the Complexity and Stability of Bacterial Community Network in Aquaculture Water Compared with the Single Addition of Either Microalgae or Probiotic

In aquatic ecosystems, particularly in aquaculture systems with high concentrations of organic particles, both FL and PA bacteria contribute to organic matter mineralization and nutrient cycling, supporting bottom-up process through interactions between microorganisms with different lifestyles [56]. Co-occurrence network analysis is widely used to elucidate the potential interaction relationships within microbial communities, providing crucial insights into the underlying ecological interactions and stability of microbial communities [57]. In the present study, we constructed integrated co-occurrence networks consisting of bacterial ASVs derived from both FL and PA communities under four treatments and compared their topological properties. All networks deviated significantly from random expectations, as indicated by differences in modularity, clustering coefficient, network diameter, and average path length. This result suggests that the detected bacterial associations were non-random and hierarchically structured.

In the present study, ASVs without clear lifestyle preferences predominated in the bacterial community networks across all treatments, accounting for 67.48–92.64% of the nodes. In the eutrophic environment, the FL and PA bacterial communities showed a high degree of overlap [58]. A study investigating the compositional characteristics of FL and PA bacterial communities in the lakeshore habitats of multiple freshwater lakes revealed that bacterial taxa without specific lifestyle preferences accounted for approximately 30% of the bacterial community networks, which was much lower than the proportions observed in our study [45]. It has been reported that suspended particles and carbon resource can promote the similarity between the FL and PA fractions [59]. In aquaculture systems, the continuous supply of feed residues, feces, and microbial flocs generates highly dynamic microhabitats with particulate matter flux, thereby blurring the boundary between FL and PA niches. Under such fluctuating and nutrient-rich conditions, bacteria with flexible lifestyles can switch between attached and free-living modes to exploit both dissolved and particulate organic substrates, conferring competitive advantages and ecological dominance. Furthermore, in the bacterial community network derived from the two treatments with microalgal addition, the proportion of ‘PAPL’ nodes was notably higher than those of treatments without microalgal addition. It meant that microalgal addition promoted the participation of taxa with particle-associated lifestyle preferences in the bacterial community network. In the two treatments with microalgal addition, furthermore, the bacterial community network exhibited denser links between nodes of the ‘PAPL’ and ‘others’ clusters in the Co_add treatment than the Alg_add treatment. Those bacterial taxa assigned without defined lifestyle preferences (i.e., ‘others’ node) probably depend on metabolites and substrates produced by the primary, fixed particle-associated bacteria, thereby functioning as secondary consumers [7,45]. These results suggest that the combined addition of microalgae and probiotic may strengthen metabolic cooperation across ecological niches, thereby accelerating particulate organic matter decomposition and nutrient cycling in aquaculture water.

The network in the Co_add treatment exhibited the highest average degree and clustering coefficient among all treatments. It also showed the lowest modularity, the smallest network diameter, and the shortest average path length. A higher average degree meant more intensive associations among microbial taxa and is considered an indicator of greater network complexity, reflecting a denser and more interactive co-occurrence network [60,61]. Furthermore, the bacterial community network showed the highest stability in the Co_add treatment, indicated by the high robustness and low vulnerability [43]. Moreover, compared with the other three treatments, the Co_add network showed a higher clustering coefficient and lower modularity. These features suggest a shift from a more modular structure to a more integrated network under the combined addition [62]. Meanwhile, a smaller network diameter also reflects a more integrated bacterial community network. Additionally, the observed lower average path length of the bacterial community network in the Co_add treatment suggested a faster exchange of information, metabolites, and energy within the microbial community [62,63]. Overall, these results suggest that the synergism of microalgae and probiotic strengthened microbial interaction, which could be important for stabilizing the bacterial community and improving ecosystem functionality in aquaculture.

### 4.3. Higher Fish Growth Performance and Better Aquaculture Water Environment Induced by the Combined Addition of Microalgae and Probiotic

The combined addition of microalgae and probiotic significantly enhanced the growth performance of largemouth bass, while single additions (Alg_add or Bac_add) showed no significant effect. Furthermore, the consistently lower TN, NH_4_^+^-N, NO_3_^−^-N, and NO_2_^−^-N concentrations were observed lower in the Co_add treatment than in the other three treatments. These improvements were likely associated with shifts in bacterial community composition and function under the Co_add treatment. Enhanced network complexity and stability may have promoted more efficient organic matter degradation and nitrogen transformation, thereby helping maintain better water quality and creating more favorable conditions for fish growth. A previous study focusing on exploring the effects of microalgae–bacteria association on fish production and water quality showed that the combined use of *Chlorella vulgaris* and *Rhodobacter azotoformans* EBL 0706 significantly improved the growth performance of the cultured Carassius auratus gibelio and reduced TN, NH_4_^+^-N, and NO_2_^−^-N concentrations of aquaculture water, which were consistent with our results [17]. Likewise, Geng et al. (2022) found that the combined application of microalgae and probiotic within a mussel–microalgae–bacteria system achieved markedly higher removal efficiencies of nitrogen, phosphorus, and organic matter than single component treatments [64].

The consistently lower TN, NH_4_^+^-N, NO_3_^−^-N, and NO_2_^−^-N concentrations in the Co_add treatment suggest more efficient nitrogen turnover. Previous studies have demonstrated that algal–bacterial synergism systems can facilitate nutrient assimilation and transformation in aquaculture water by coupling autotrophic uptake and heterotrophic mineralization compared to single microalgal or probiotic inoculation [30,65,66]. In the present study, we found that bacterial taxa of FL communities significantly enriched in the Co_add treatment were associated with carbon and nitrogen cycling processes such as nitrogen respiration, nitrate respiration, cellulolysis, methylotrophy, methanol oxidation, ureolysis, and nitrate reduction. The enrichment of these heterotrophic bacterial taxa likely promoted the mineralization of organic nitrogen and accelerated nitrogen turnover, thereby reducing the accumulation of toxic nitrogenous compounds in the aquaculture water. The improved water quality could in turn have contributed to the better growth performance of largemouth bass under the Co_add treatment. Lower concentrations of ammonia and nitrite reduce metabolic stress and energy expenditure for detoxification, allowing more energy to be allocated to growth [67].

Microalgae and probiotics have long been widely applied in aquaculture as a feed supplement, growth promoter, and immunostimulant [9,11]. However, studies investigating microalgal–probiotic associations as a microbial management approach in *Micropterus salmoides* culture remain scarce. The present findings provide empirical evidence that synergistic microbial assemblages can enhance nutrient utilization efficiency and biological performance at the system level, underscoring the functional complementarity between photosynthetic microalgae and heterotrophic bacteria in improving aquaculture efficiency [15,68].

Despite these promising results, the current study still has limitations. Due to the limited sample size, the mesocosm-based experimental design, and the relatively short experimental duration, it was not possible to establish a robust causal model linking the observed shifts in bacterial community composition and function with water quality improvement and fish growth performance following microalgal and bacterial addition. In addition, the controlled mesocosm setting and limited temporal scale may constrain the extrapolation of our findings to broader aquaculture conditions. Moreover, because the community analysis was based on 16 S rRNA amplicon sequencing, the ASV dataset did not provide reliable strain-level resolution to distinguish the exogenously added *Bacillus subtilis* from closely related indigenous Bacillus populations. Future studies should aim to validate these patterns across broader spatial and temporal scales and use strain-specific or higher-resolution genomic approaches to better trace the added probiotic strain and further elucidate the underlying microbial ecological mechanisms.

## 5. Conclusions

In this study, we examined the effects of single and combined additions of microalgae (*Chlorella vulgaris*) and probiotic (*Bacillus subtilis*) on the growth performance, water quality, bacterial community structure, and co-occurrence networks in *Micropterus salmoides* aquaculture. Overall, the treatment with combined addition exhibited the highest fish growth performance and the lowest concentrations of total nitrogen and inorganic nitrogen in the aquaculture water. The compositions and functions of bacterial communities were significantly altered by microalgal and probiotic addition, with distinct responses in the FL and PA fractions. Different key contributors to water quality variations were identified between the two bacterial lifestyles. Furthermore, the combined addition resulted in integrated network structure with increased network complexity and stability, suggesting higher bacterial community resilience. These results reveal that microalgae–probiotic synergism alters bacterial community compositions, potential functions, and co-occurrence network patterns of bacterial communities, thereby promoting system resilience and ecological stability in aquaculture environments. These findings also highlight the potential of the combined addition of microalgae and probiotic as a sustainable approach to maintain water quality, enhance fish growth, and stabilize microbial communities in intensive aquaculture.

## Figures and Tables

**Table 1:** Effects of different treatments on growth performance of *Micropterus salmoides*. CK, control treatment; Alg_add, treatment with single addition of microalgae; Bac_add, treatment with single addition of probiotic; Co_add, treatment with combined addition of microalgae and probiotic. Different letters indicate significant differences across treatments (*p* < 0.05). Values are presented as mean ± SD (n = 3 per treatment).

Treatment	Initial Body Weight (g)	Terminal Body Weight (g)	Weight Gain Rate (%)	Specific Growth Rate (%·d^−1^)
CK	11.79 ± 0.71 ^a^	56.64 ± 12.13 ^b^	380.21 ± 97.43 ^b^	2.78 ± 0.41 ^b^
Alg_add	11.87 ± 0.84 ^a^	63.39 ± 16.34 ^ab^	439.27 ± 158.56 ^ab^	2.99 ± 0.44 ^ab^
Bac_add	11.61 ± 1.16 ^a^	65.06 ± 20.32 ^ab^	461.02 ± 168.23 ^ab^	3.02 ± 0.47 ^ab^
Co_add	11.58 ± 0.89 ^a^	69.42 ± 20.40 ^a^	503.09 ± 182.61 ^a^	3.13 ± 0.47 ^a^

**Table 2:** Effects of lifestyle, treatment, time point, and their interactions on bacterial alpha diversity and community dissimilarity, assessed using three-way ANOVA and permutational multivariate analysis of variance (PERMANOVA).

	Alpha Diversity		Community Dissimilarity	
	Richness	Evenness	Bray–Curtis	
	F Value	F Value	R^2^	F Value
Lifestyle	42.8 ***	49.6 ***	0.12	7.5 ***
Treatment	0.5	1.7	0.07	1.4 *
Time point	1.4	0.5	0.11	6.5 ***
Lifestyle: Treatment	0.8	0.8	0.05	1.1
Lifestyle: Time point	2.2	0.8	0.06	3.5 ***
Treatment: Time point	0.7	0.4	0.04	0.8
Lifestyle: Treatment: Time point	0.1	0.2	0.03	0.6

* *p* < 0.05; *** *p* < 0.001.

**Table 3:** Topological properties of the empirical network and associated random network for bacterial communities containing both lifestyles derived from different treatments. CK, control treatment; Alg_add, treatment with single addition of microalgae; Bac_add, treatment with single addition of probiotic; Co_add, treatment with combined addition of microalgae and probiotic.

	Network Indices	CK	Alg_add	Bac_add	Co_add
Empirical network	No. of nodes	303	286	326	326
	No. of edges	1652	1041	2157	3160
	Average degree	10.9	7.28	13.23	19.39
	Modularity	0.359 ^a^	0.351 ^a^	0.322 ^a^	0.244 ^a^
	Clustering coefficient	0.180 ^a^	0.144 ^a^	0.183 ^a^	0.232 ^a^
	Network diameter	6.52 ^a^	7.39 ^a^	5.99 ^a^	5.89 ^a^
	Average path length	3.24 ^a^	3.82 ^a^	3.12 ^a^	2.81 ^a^
Random network	Modularity	0.262 (0.005)	0.336 (0.007)	0.233 (0.005)	0.185 (0.004)
	Clustering coefficient	0.036 (0.002)	0.026 (0.003)	0.041 (0.002)	0.060 (0.002)
	Network diameter	4.22 (0.42)	5.59 (0.52)	4.01 (0.07)	3.21 (0.41)
	Average path length	2.65 (0.003)	3.06 (0.008)	2.53 (0.002)	2.24 (0.002)

^a^ Significant difference between the empirical network and the random network (*p* < 0.001, Z-test).

## Data Availability

The raw data are accessible in NODE (https://www.biosino.org/node) through the URL: https://www.biosino.org/node/experiment/detail/OEX00032700 (accessed on 3 November 2025).

## Supplementary Materials

The following supporting information can be downloaded at https://www.mdpi.com/article/10.3390/biology15070566/s1, Figure S1: Schematic diagram of the experimental design and sampling timeline. Figure S2: Relative abundance of the top 10 dominant bacterial phyla in the free-living (FL, a) and particle-associated (PA, b) communities across the four treatments. Phyla were ranked according to their overall mean relative abundance across all samples, and only the top 10 phyla are shown individually; all remaining phyla are grouped as Others. CK, control treatment; Alg_add, treatment with single addition of microalgae; Bac_add, treatment with single addition of probiotic; Co_add, treatment with combined addition of microalgae and probiotic. Figure S3: Relative abundance of the top 10 dominant bacterial genera in the free-living (FL, a) and particle-associated (PA, b) communities across the four treatments. Genera were ranked according to their overall mean relative abundance across all samples, and only the top 10 genera are shown individually; all remaining phyla are grouped as Others. CK, control treatment; Alg_add, treatment with single addition of microalgae; Bac_add, treatment with single addition of probiotic; Co_add, treatment with combined addition of microalgae and probiotic. Table S1: The influences of lifestyle, treatment, time point and their interactions on alpha diversity and community dissimilarity of bacterial communities using three-way ANOVA test and permutational multivariate analysis of variance (PerMANOVA).
