# Supplementary material for: Combined Addition of Microalgae and Probiotic Enhances Bacterial Community Network Stability, Water Quality, and Fish Growth in Micropterus salmoides Aquaculture
Source: Biology (Basel). 2026 Apr 1;15(7):566. doi: 10.3390/biology15070566 (PMC13072078; doi:10.3390/biology15070566)
Supplement: Supplementary file 1 [file biology-15-00566-s001.zip › Table S1.pdf]

Table S1 The influences of lifestyle, treatment, timepoint and their interactions on alpha diversity and community dissimilarity of bacterial communities using three-way ANOVA test and permutational multivariate analysis of variance (PerMANOVA).

	**Alpha diversity**		**Community dissimilarity**	
	**Richness**	**Evenness**	**Bray Curtis**	
	***F* value**	***F* value**	$\mathbf{R^2}$	***F* value**
**Lifestyle**	**42.8*** **	**49.6*** **	0.12	**7.5*** **
**Treatment**	0.5	1.7	0.07	**1.4***
**Timepoint**	1.4	0.5	0.11	**6.5*** **
**Lifestyle: Treatment**	0.8	0.8	0.05	1.1
**Lifestyle: Timepoint**	2.2	0.8	0.06	**3.5*** **
**Treatment: Timepoint**	0.7	0.4	0.04	0.8
**Lifestyle: Treatment: Timepoint**	0.1	0.2	0.03	0.6

*P < 0.05; **P < 0.01; ***P < 0.001
