# Supplementary figures and images for: Combined Addition of Microalgae and Probiotic Enhances Bacterial Community Network Stability, Water Quality, and Fish Growth in Micropterus salmoides Aquaculture
Source: Biology (Basel). 2026 Apr 1;15(7):566. doi: 10.3390/biology15070566 (PMC13072078; doi:10.3390/biology15070566)

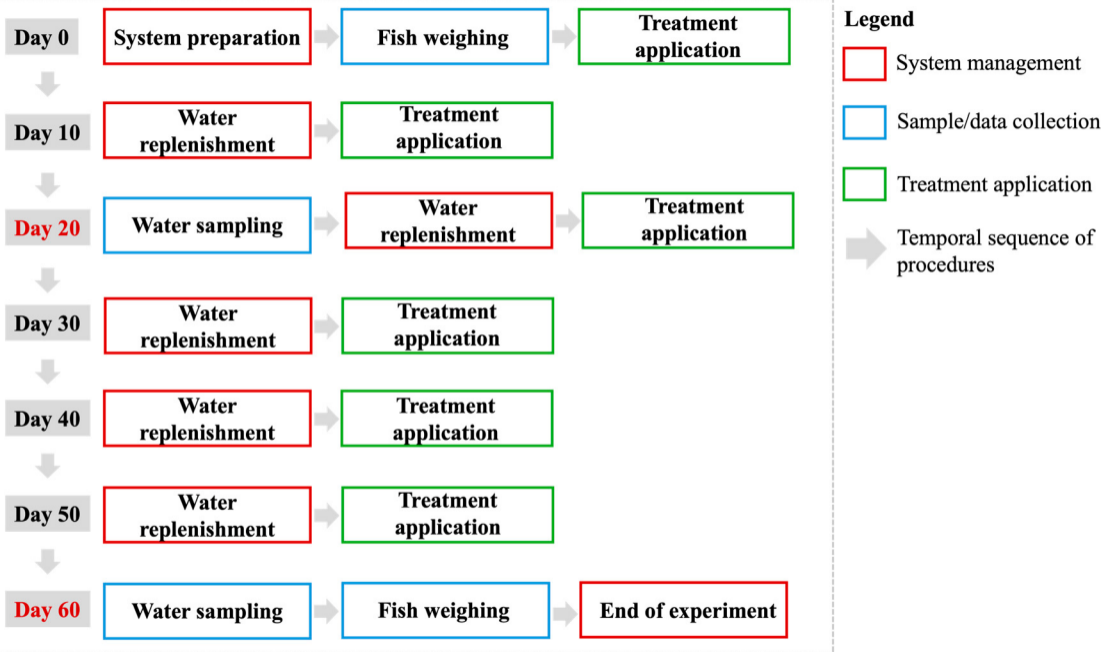

Supplement: Supplementary file 1 [file biology-15-00566-s001.zip › Figure S1.pdf]

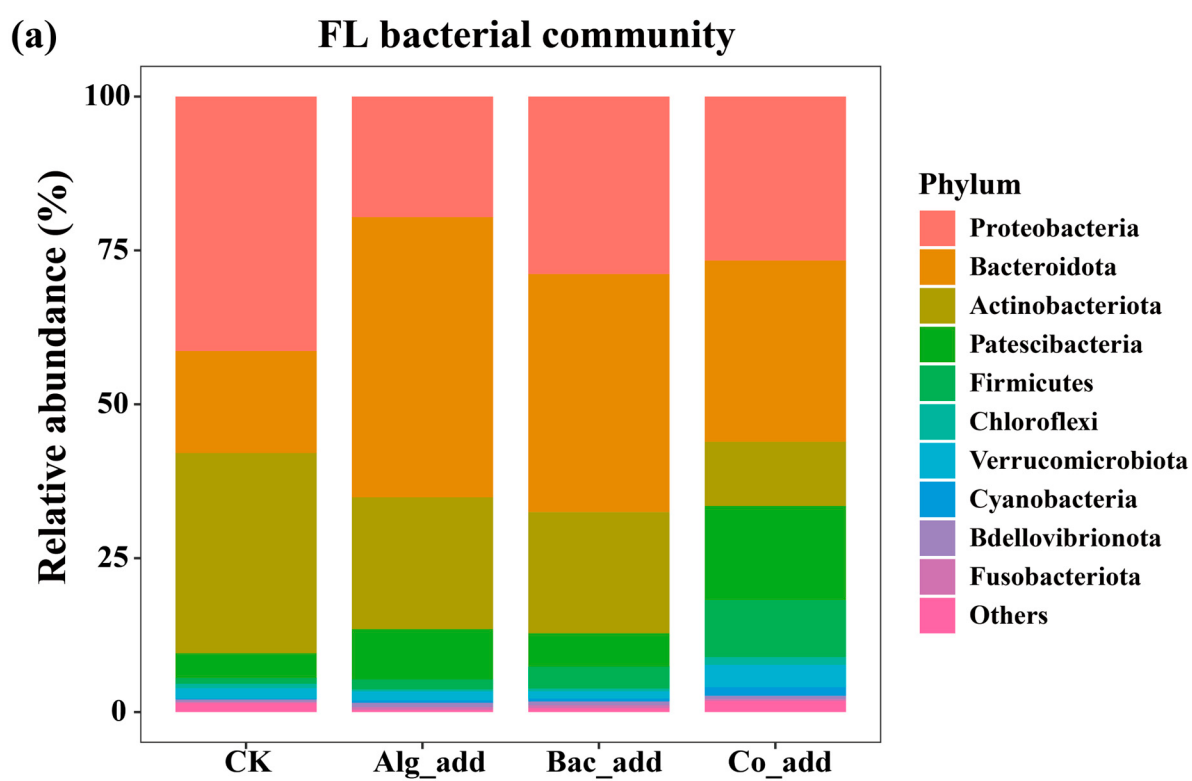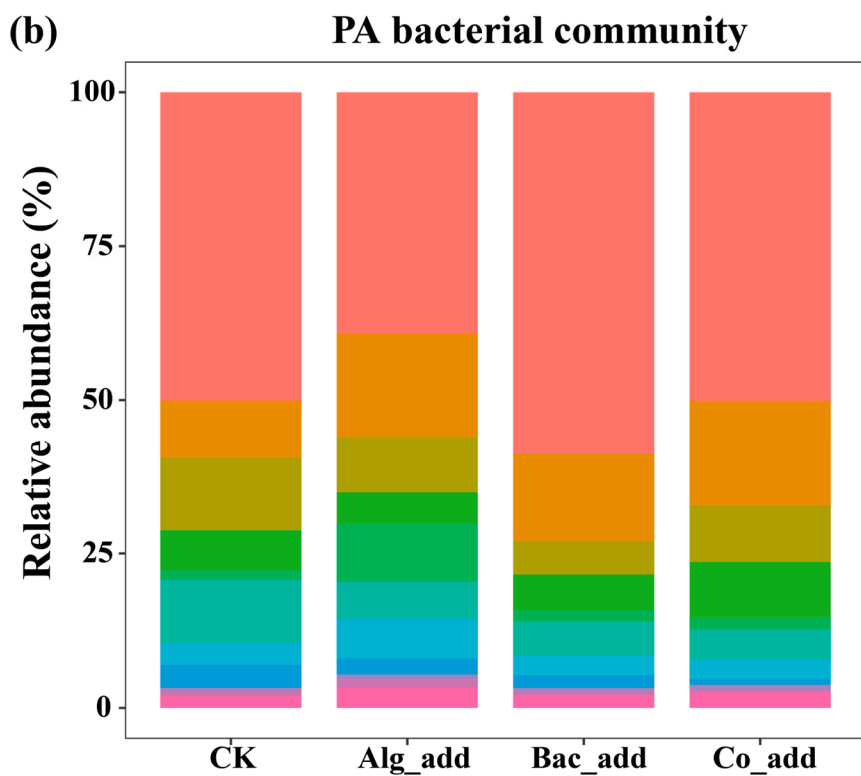

Supplement: Supplementary file 1 [file biology-15-00566-s001.zip › Figure S2.pdf]

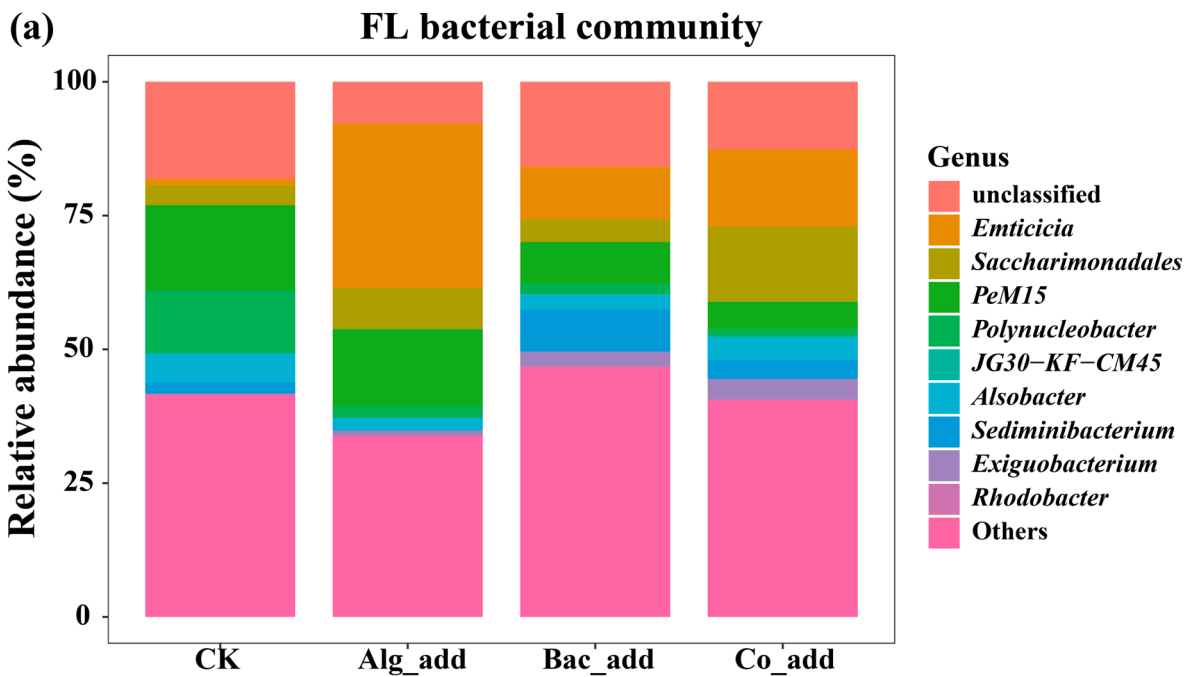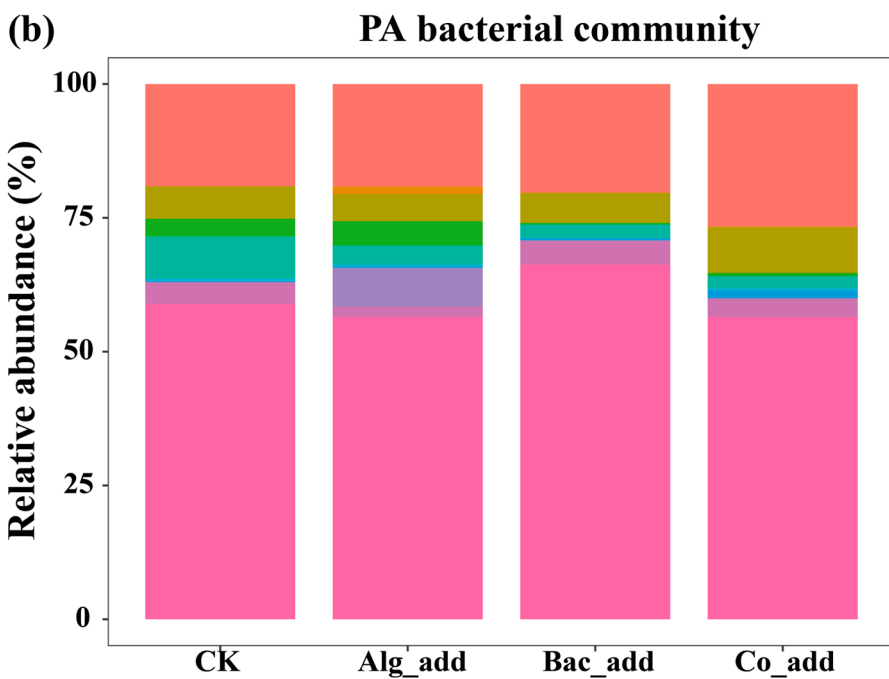

Supplement: Supplementary file 1 [file biology-15-00566-s001.zip › Figure S3.pdf]
